# Memfractance of Proteinoids

**DOI:** 10.1021/acsomega.3c09330

**Published:** 2024-03-18

**Authors:** Panagiotis Mougkogiannis, Andrew Adamatzky

**Affiliations:** Unconventional Computing Laboratory, UWE, Bristol BS16 1QY, U.K.

## Abstract

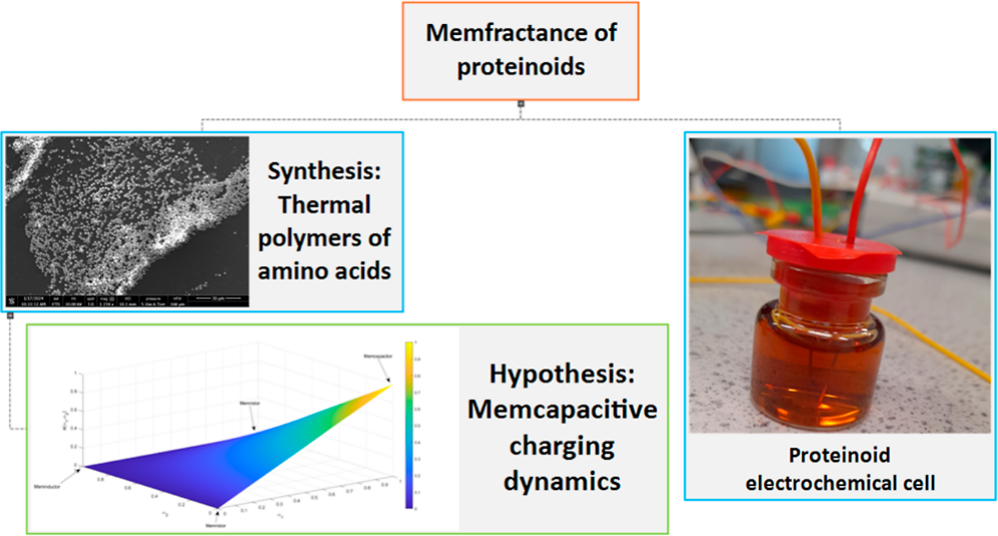

Proteinoids, or thermal proteins, are amino acid polymers formed at high temperatures by nonbiological processes. The objective of this study is to examine the memfractance characteristics of proteinoids within a supersaturated hydroxyapatite solution. The ionic solution utilized for the current–voltage (*I*–*V*) measurements possessed an ionic strength of 0.15 mol/L, a temperature of 37 °C, and a pH value of 7.4. The *I*–*V* curves exhibited distinct spikes, which are hypothesized to arise from the capacitive charging and discharging of the proteinoid–hydroxyapatite media. The experimental results demonstrated a positive correlation between the concentration of proteinoids and the observed number of spikes in the *I*–*V* curves. This observation provides evidence in favor of the hypothesis that the spikes originate from the proteinoids’ capacitive characteristics. The memfractance behavior exemplifies the capacity of proteinoids to retain electrical charge within the hydrated hydroxyapatite media. Additional investigation is required in order to comprehensively identify the memcapacitive phenomena and delve into their implications for models of protocellular membranes. In a nutshell, this study provides empirical support for the existence of capacitive membrane-memfractance mechanisms in ensembles of proteinoids.

## 1 Introduction

Proteinoids, or thermal proteins, are molecular assemblies that resemble proteins and were initially synthesized in the 1960s by Sidney Fox using thermal polycondensation of amino acids.^1,2^ The synthetic polypeptides have the remarkable ability to self-organize into microspheres that closely resemble structures found in cells. Proteinoids exhibit various fascinating properties, such as catalytic activity, information storage and transfer, and evolutionary behavior. Their capacity to self-organize and self-replicate under certain environmental conditions offers clues into the protobiological processes that may have preceded cellular life.^3−5^ While far less complex than modern cellular systems that arose from billions of years of biological evolution, these properties offer clues into key functions that are necessarily emergent in the transition from prebiotic chemistry to life.^1,2,6−10^

Proteinoids have been a subject of study for many years, but only in 2021, have they been proposed as candidates for future neuromorphic computers,^11^ and just recently, research has started to reveal their potential for electrical signaling and memory functions.^12−14^ When experimenting with proteinoids, we found that their bulk aggregates, formed from many individual proteinoid particles in solution, exhibit a wide range of unusual electrical properties that could be used for bioinspired electronics and computing. To shed more light on these properties, we have investigated the memfractive, i.e., showing the combined effects of memory resistive and capacitive phenomena,^15^ behavior of proteinoids suspended as a hydrogel in supersaturated solutions of calcium phosphates at 7.4 pH, an ionic strength of 0.15 mol/L, and a temperature of 37 °C.^16^ We demonstrated that proteinoids exhibit pinched hysteresis loops in current–voltage curves, which are distinctive features of the memfractance systems. The presence of more hysteresis loops is observed as the proteinoid concentration increases, suggesting that the memfractance is a result of the proteinoids themselves. The memfractance properties that we propose stem from the proteinoids’ ability to store charge in their hydration layers, functioning as primitive capacitors. Details of the methodological setup and our discoveries follow.

Memristive systems demonstrate a unique property in which their resistance retains a memory of past electrical inputs, which is then linked to their current output. This characteristic holds great potential for the development of bio-inspired computing. Proteinoids, serving as analogues of prebiotic proteins, have demonstrated self-organized characteristics resembling those of living organisms, which could potentially be applied to the development of devices. Nevertheless, the incorporation of proteinoids into operational memristor structures has hardly been investigated. Thus, the objective of this study was to create and analyze memristive proteinoids in order to accurately evaluate their ability to store memory and perform nonlinear dynamic processing. We postulated that proteinoids feature intrinsic biophysical characteristics that can be translated into observable memfractance behaviors using electrical analysis. This work uniquely establishes reliable standards and dynamic models to clarify proteinoids as components of bio-computing memory. In addition to their application in computing, the use of proteinoids can offer valuable insights into the beginnings of innate information processing in early biomolecular systems that existed before cellular life emerged.

## 2 Materials and Methods

l-Phenylalanine, l-aspartic acid, l-histidine, l-glutamic acid, and l-lysine were purchased from Sigma-Aldrich, ensuring a purity level over 98%. The thermal polycondensation approach developed by Mougkogiannis et al.^17^ was employed to synthesize proteinoids. In brief, equimolar blends of the amino acids were subjected to heating at a temperature of 180 °C, with continuous stirring and under a nitrogen atmosphere, for a duration of 30 min. The proteinoids obtained were subjected to lyophilization and thereafter kept at room temperature. The morphology of proteinoids was analyzed through the use of scanning electron microscopy (SEM) with the aid of an FEI Quanta 650 microscope. This examination was conducted under conditions of high vacuum subsequent to the application of gold sputter-coating. The pH measurements were performed utilizing a Cole Parmer silver/silver chloride (Ag/AgCl) glass electrode that was calibrated with standard buffer solutions prior to each use.^18^ The electrode was linked to a MADGETECH pHTemp2000 data logger in order to capture and store pH data over a period of time in a CSV format.

The *I*–*V* characteristics were measured by employing a Keithley 2450 sourcemeter. The subdermal needle electrodes used were made of platinum–iridium-coated stainless steel, specifically obtained from Spes Medica S.r.l. in Italy. These electrodes were placed in the proteinoids, maintaining an approximate separation of 10 mm between each pair of electrodes. We finally utilized a high-resolution data logger equipped with a 24-bit analog-to-digital converter (ADC-24, Pico Technology, UK) to accurately record the electrical activity of the proteinoids.

The chemicals used for the experiment included potassium dihydrogen phosphate, potassium nitrate, and calcium nitrate tetrahydrate. The potassium dihydrogen phosphate was obtained from Fischer Chemical, with a minimum purity of 99.5%, a molecular weight of 136.09 g/mol, and a CAS number of 7778-77-0. The potassium nitrate was purchased from Sigma-Aldrich, with a minimum purity of 99.0%, a molecular weight of 101.10 g/mol, and a CAS number of 7757-79-1. The calcium nitrate tetrahydrate, with a molecular weight of 236.15 g/mol and CAS number 13477-34-4, was acquired from Thermo Scientific. The chemicals employed in the experiment were utilized in their original form without undergoing any additional purification procedures. The potassium dihydrogen phosphate, potassium nitrate, and calcium nitrate tetrahydrate used in the experiment were of analytical quality.

The prepared calcium phosphate supersaturated exceeded the mineral solubility needed for crystallization to occur before adding proteinoids, specifically at over 11 times saturation for hydroxyapatite (HAP), around 0.2 times saturation for octacalcium phosphate (OCP), and roughly saturation level for brushite. To relate to this, a saturation level of 1 allows mineral and solution equilibration, while higher values denote supersaturation, enabling further mineral growth, as was utilized here.

The solutions were prepared by combining calcium, phosphate, and hydroxide in a molar ratio of 10:6:2 within a 200 mL vessel. This resulted in an ionic strength of 0.15 mol/L at a temperature of 37 °C and a pH of 7.4. Following the production of the supersaturated calcium phosphate solutions, a total of 180 mg of proteinoids were introduced into the reaction vessel. The HAP’s considerably high σ value suggests that the solutions were significantly supersaturated with HAP before the addition of proteinoids. Additional measurements will be conducted to evaluate the influence of proteinoids on the speciation of calcium phosphate and the kinetics of precipitation under the given conditions.

The solubility of calcium phosphate minerals is determined by their Ca/P molar ratio and crystal structure. Table 1 presents the solubility data that have been measured for various significant calcium orthophosphate compounds, such as HAP and OCP. The solubility of a substance is typically represented by the negative logarithm of its solubility product (*K*_sp_), which is determined at temperatures of 25 and 37 °C. A lower value of −log(*K*_sp_) indicates a lower solubility. As shown in Table 1, the lowest solubility of OCP has −log(*K*_sp_) values of 98.6 and 117.2 at 25 and 37 °C, respectively. On the other hand, brushite (CaHPO_4_·2H_2_O) exhibits significantly higher solubility. The insoluble nature of OCP makes it a significant precursor phase for the formation of biological apatite. The table presented here serves as a valuable resource for understanding the relative solubilities of important calcium phosphate mineral phases under physiological conditions. The solubility products exhibit a wide range of variabilities, indicating that the speciation of calcium phosphate is greatly influenced by the Ca/P ratio and crystallinity. The Visual MINTEQ version 4 software was used to perform chemical speciation modeling and calculate the relative supersaturation values (σ). The supersaturated calcium phosphate solutions at 25 and 37 °C were modeled using the temperature-dependent solubility products and equilibrium constants found in the Visual MINTEQ database.^19^

**Table 1:** Ca/P Molar Ratios, Chemical Formulas, and Solubilities of Some Calcium Orthophosphate Minerals^18^

Ca/P molar ratio	chemical formula	solubility (−log(*K*_sp_))		
		25 °C	37 °C	37 °C (solubility product)
1.00	CaHPO_4_·2H_2_O	6.59	6.73	1.87 × 10^–7^ M^2^
1.00	CaHPO_4_	6.90	6.04	9.2 × 10^–7^ M^2^
1.33	Ca_8_(HPO_4_)_2_(PO_4_)_4_·5H_2_O	96.6	98.6	2.5 × 10^–99^ M^16^
1.20–2.20	C_a*x*_H_*y*_(PO_4_)_*z*_·*n*H_2_O			
1.50	α-Ca_3_(PO_4_)_2_	25.5	28.5	2.8 × 10^–29^ M^5^
1.50	β-Ca_3_(PO_4_)_2_	28.9	29.6	2.5 × 10^–30^ M^5^
1.67	Ca_10_(PO_4_)_6_(OH)_2_	116.8	117.2	5.5 × 10^–118^ M^18^
1.67	Ca_10_(PO_4_)_6_F_2_	120.0	122.3	5.0 × 10^–123^ M^18^

The relative supersaturation (σ) of a mineral can be expressed as
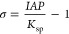
1where *IAP* is the ion activity product and *K*_sp_ is the solubility product. The supersaturation ratio *S* is then defined as
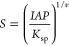
2where *v* is the number of ions in the mineral’s formula unit. For HAP, the *IAP* is given by
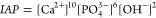
3When *S* = 1, the solution is at equilibrium with the mineral. For *S* < 1, the solution is undersaturated, and the mineral dissolves. When *S* > 1, the solution is supersaturated, and the mineral precipitates. Values of σ and *S* are useful indicators of the saturation state of a mineral-solution system.

## 3 Results

### 3.1 SEM of HAP–Proteinoid Microstructures

The use of SEM facilitated the identification of proteinoid–HAP and proteinoid–calcium phosphate composites with a high level of detail. The porous, brain-like shape of the proteinoid–HAP matrix is illustrated in Figure 2. Within this structure, the spherical proteinoid nanospheres, measuring ca. 99 nm in diameter, are closely integrated within the gaps and pores of the HAP framework. The previous observation presents empirical support for the consistent integration of proteinoids into the intricate topographical structure of the HAP scaffolds. In the Appendix, Figure 14 displays the energy-dispersive X-ray (EDX) spectrum of proteinoids.

**Figure 1:**
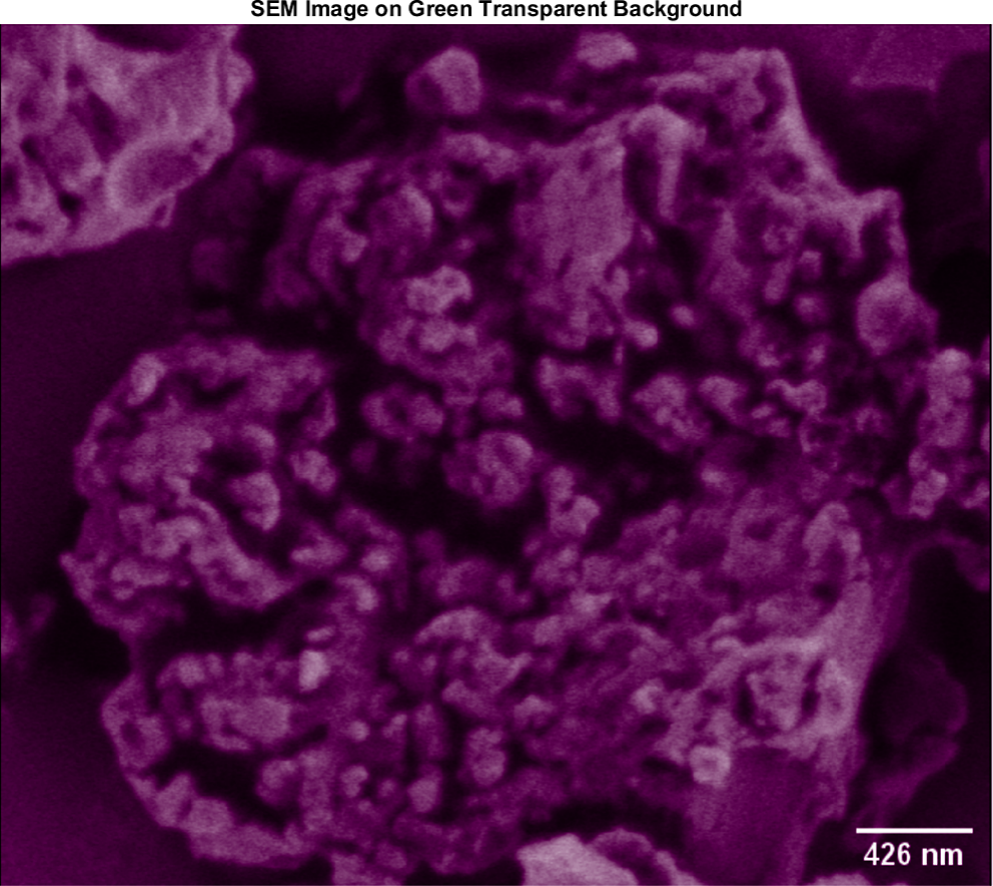
Low-magnification SEM image shows spherical proteinoid nanospheres that are integrated within the HAP matrix. Each proteinoid has a diameter of 99 nm. The proteinoids are found embedded within pores and cracks throughout the HAP structure. The SEM images were obtained using an accelerating voltage of 1.50 kV and a magnification of ×25,000. The scale bar in the image measures 426 nm. The results show that proteinoid nanospheres are closely integrated into the intricate topological structure of the HAP scaffolds.

**Figure 2:**
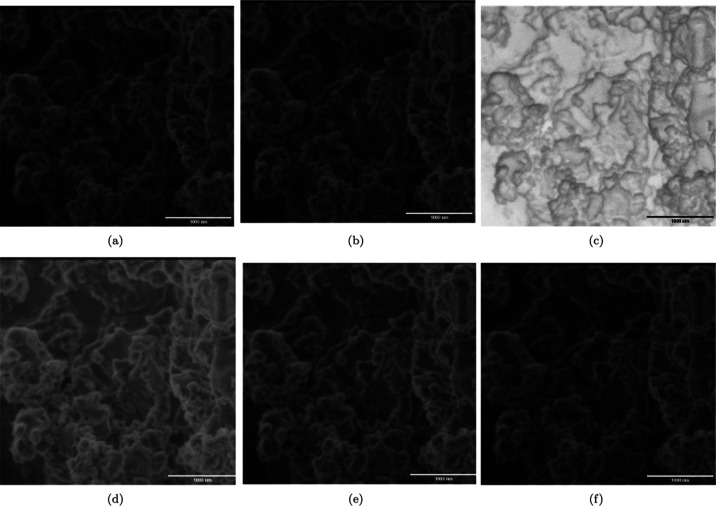
SEM image of l-Glu:l-Arg proteinoids-stabilized OCP/dicalcium dihydrogen phosphate particles. (a) SEM micrograph of the stabilized calcium phosphate particle plate and blade morphology. There are visible blades up to 308.562 nm in length. (b) Image with no filtering. (c) Negative picture. (d) Image filtered with gamma = 0.5. (e) Image filtered with gamma = 0.8. (f) Image filtered with gamma = 1.0. SEM imaging was carried out at a voltage of 1.30 kV, a magnification of 25,000×, a spot size of 2.0, and a scale bar of 426 nm. The findings show that proteinoids may modulate the crystallization of calcium phosphate species into anisotropic microstructures. Image processing improves contrast and focuses attention to morphological details.

Furthermore, Figure 1 illustrates the structural characteristics of the blade and plate-like formations achieved through the use of proteinoids for the stabilization of OCP and dicalcium dihydrogen phosphate granules. In the presence of l-Lys:l-Phe:l-His:PLLA proteinoids, the calcium phosphate blades exhibited lengths of up to 308 nm. The application of image processing techniques resulted in the improvement of contrast and morphological information related to the anisotropic structures. In general, the microscopy results validate the capacity of proteinoids to facilitate the formation of well-defined geometries in the crystallization process of biocompatible calcium phosphates.

The results obtained from SEM reveal that proteinoids have a significant impact on the morphological aspects involved in the process of calcium phosphate crystallization. Despite the pH being kept above 7.4, it is probable that statistical fluctuations facilitated the temporary development of embryonic calcium phosphate phases through the Ostwald ripening mechanism. Within this particular mechanism, the dissolution of smaller nuclei occurs, followed by their subsequent redeposition onto larger particles.

According to the Ostwald rule of stages, it is observed that in the process of crystallization, the initial phases to crystallize are often those with lower thermodynamic stability, whereas subsequent phases exhibit progressively higher stability. This particular kinetic phenomenon can be mathematically described by the change in free energy during a phase transition.^20,21^
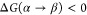
4where α is the less stable phase and β is the more stable phase.
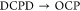
5
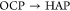
6Since Δ*G* < 0 for the above reactions, HAP is the most thermodynamically favorable end product. However, the high kinetic barriers allow persistence of the intermediate OCP and DCPD (dicalcium dihydrated phosphate—brushite) phases. In general, the Ostwald ripening mechanism facilitates crystal growth by passing thermodynamically unstable intermediates under the control of kinetics until the final stable phase is reached in accordance with equilibrium thermodynamics.

The proteinoids seem to interact with the growing calcium phosphate molecules in solution. They adhere to them and act as templates, shaping them into anisotropic blades and plates. This can happen when charged groups on proteinoids bind to ionic sites on the calcium phosphate surfaces. Conformational matching between proteinoids and crystal facets can also play a role in directing the oriented growth. Moreover, the presence of proteinoids bound to molecules can potentially impact solubility equilibria and influence the rate of growth kinetics.

Proteinoids can influence and direct the growth mechanisms, architectures, and resulting morphologies of calcium phosphate crystals during directed crystallization, as evidenced by the changes observed compared with abiotic control samples. This mechanism enables biologically directed mineralization by using proteinoid interfaces to template and regulate the formation of minerals from temporary precursor phases.

Elemental analysis via EDX spectroscopy provided deeper insight into the molecular composition and uniformity of the synthesized proteinoid microspheres. As displayed in the supplementary EDX map (AppendixFigures 14 and 15), l-glutamic acid:l-phenylalanine (l-Glu:l-Phe) proteinoids exhibit spatially consistent carbon, nitrogen, and oxygen components in line with formulation stoichiometry.

### 3.2 Aqueous vs Mineralized Proteinoid Memfractance

#### 3.2.1 Memfractance Properties of the l-Lys:l-Phe:l-His:PLLA and l-Glu:l-Phe:PLLA Proteinoid Systems

**Figure 3:**
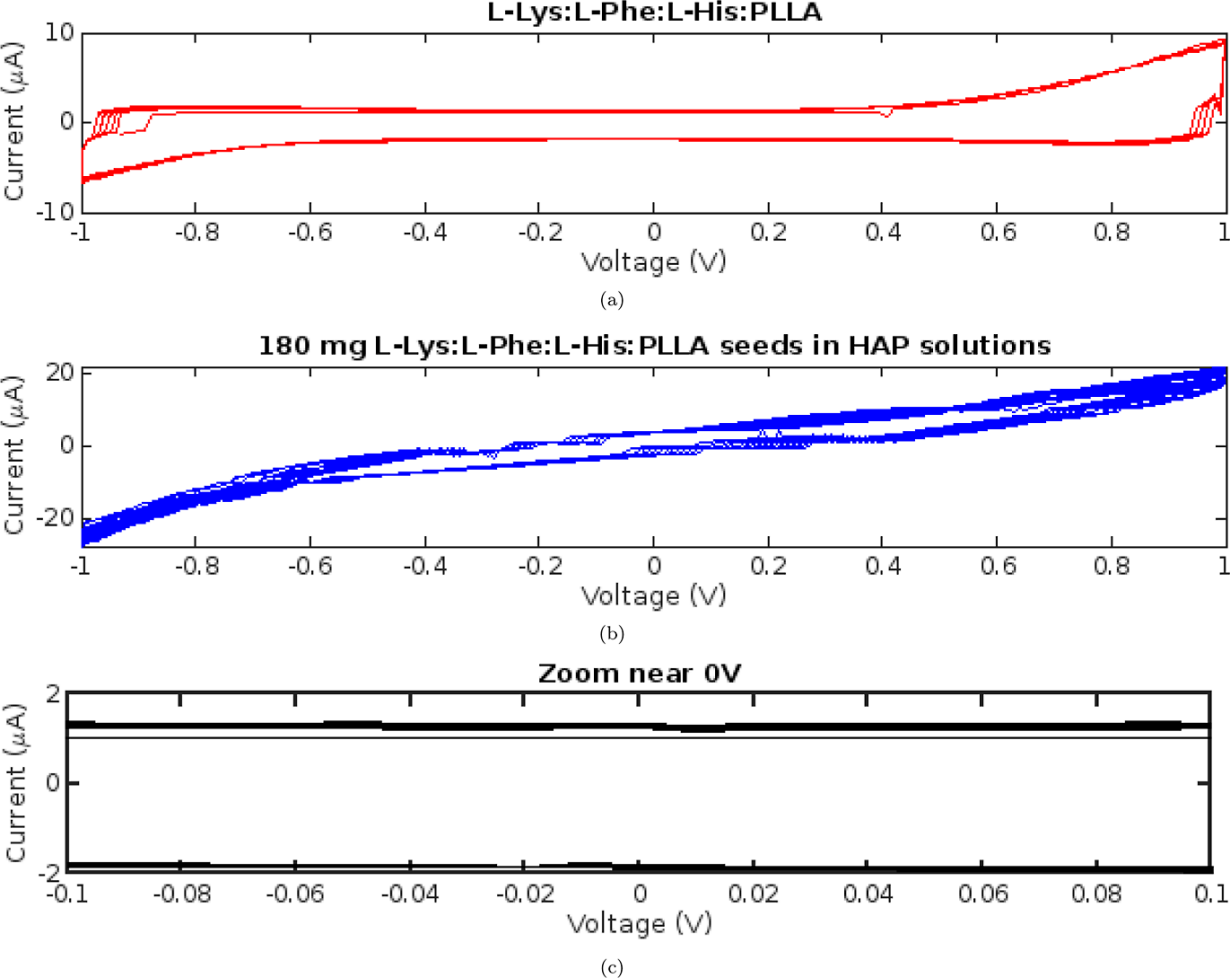
Curves of current–voltage (*I*–*V*) for suspensions of proteinoids. (a) Proteinoids diluted in water exhibit a hysteresis loop with a peak current of approximately 10 μA. (b) Adding 180 mg of proteinoids to a 200 mL supersaturated HAP solution doubles the maximal current to 20 μA. (c) The narrowed hysteresis loops approach closer to a linear current–voltage relationship around 0 V upon introducing the HAP solution, signifying a potential shift from broader memfractance behaviors toward more dominant memristive conduction mechanisms in the proteinoid–mineral mixtures. Increased current and altered pinching suggest that proteinoids interact with supersaturated calcium phosphates, thereby enhancing conductivity and electrical memory effects.

The current–voltage characteristics of the proteinoid suspensions were investigated by applying a full voltage sweep cycle. As depicted in Figure 3a, a voltage ranging from −1 to +1 V and back was applied across the samples. The resulting current was measured throughout the voltage cycle to obtain the complete current–voltage (*I*–*V*) profile. The addition of proteinoids like l-Lys:l-Phe:l-His:PLLA to the supersaturated HAP solutions resulted in a nearly 100% increase in the maximal measured current in the *I*–*V* curves, from approximately 10 to 20 μA (Figure 3b). This doubling of the current suggests that the proteinoids have increased conductivity, which can be attributed to enhanced ion mobility. Proteinoids presumably provide an additional surface area for charge carrier migration and transport. With the introduction of HAP, the hysteresis loop shape became narrower and less rounded near the 0 V midpoint, approaching a more linear current–voltage relationship compared to the prominent curved hysteretic behavior without HAP (Figure 3c). The decrease in loop constriction indicates a transition from pure memfractance behavior to memristance behavior. This suggests that calcium phosphates modulate the capacitive effects of proteinoids. It appears that the HAP interacts with proteinoid assemblies, altering their capacity to store charge. These pronounced shifts in the *I*–*V* profile upon addition of supersaturated HAP indicate that the proteinoids in these solutions are not electrically inert. Almost certainly, the proteinoids engage in specific interactions with the calcium phosphate species. To elucidate the precise mechanism, be it direct surface adsorption, complexation, or conformational changes in the proteinoids, additional characterization is required. Nevertheless, these preliminary *I*–*V* measurements reveal that proteinoids in HAP solutions exhibit improved conductivity and modified memfractance behaviors.

**Figure 4:**
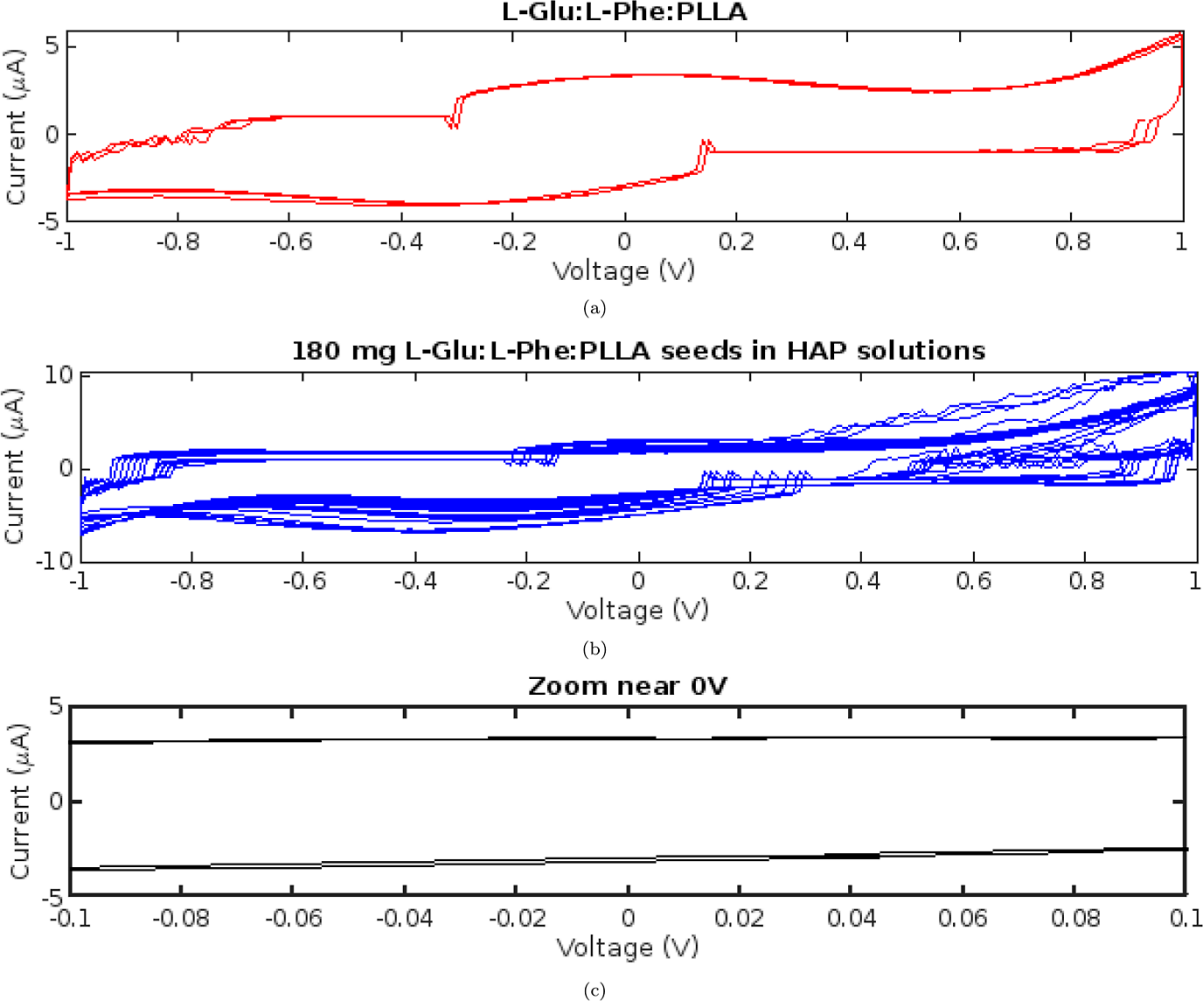
Curves of current–voltage (*I*–*V*) for suspensions of proteinoids. (a) l-Glu:l-Phe:PLLA proteinoids dissolved in water exhibit a hysteresis loop for the *I*–*V* response. (b) The addition of 180 mg of l-Glu:l-Phe proteinoid seeds to a supersaturated HAP solution increases the current and modifies the morphology of the pinched hysteresis near 0 V. (c) Enlarged view of the *I*–*V* curves near 0 V. Comparing current–voltage traces for aqueous proteinoids (a) and with added HAP (b) indicates subtle differences, with the latter showing slightly more ohmic-dominant character. However, quantifying tiny hysteresis looping changes on the order of 1–5% proves difficult as all tracings crossover near-origin within error bounds. Changes suggest that proteinoids are interacting with calcium phosphates to improve conductivity.

The maximal current for l-Glu:l-Phe:PLLA proteinoids dissolved in water was approximately 5 μA (Figure 4a). The current doubled to 10 μA when 180 mg of the same proteinoid composition was added to a supersaturated HAP solution (Figure 4b). This doubling of the current suggests that the proteinoids interact with the calcium phosphates in solution, most likely by adsorption to surfaces or insertion into the hydration layers. The proteinoids appear to provide additional ion migration pathways, thereby enhancing conductivity. As has been observed with other proteinoid compositions, the HAP additive enables enhanced memristive and memcapacitive behaviors.

In the Appendix, Figure 15 displays the current–voltage characterization of (a) proteinoid (l-Glu:l-Phe:l-His:PLLA) memristive behavior and (b) proteinoid in HAP over time. The *x*-axis represents time in seconds (s). The measured current (*I*) is presented on the left *y*-axis in units of microamps (μA). The applied input voltage (*V*) is indicated on the right *y*-axis in volts (Volts).

The increase in conductivity observed with proteinoids can be attributed to the formation of a composite matrix consisting of HAP and proteinoids. This matrix helps facilitate the movement of the ions. Previous studies have demonstrated that HAP can create composites with collagen, allowing for anhydrous proton conduction without the need for external humidification.^22^ The proteinoids are believed to function as a scaffold that mimics collagen, thereby improving the movement of protons within the HAP medium.

Furthermore, it has been reported that proteins can alter the crystal structure and morphology of HAP during the process of mineralization.^23−26^ Proteinoids have the ability to stimulate the formation of smaller HAP crystals that exhibit higher crystallinity and a larger surface area. These features have the potential to decrease the grain boundary resistance and enhance the charge carrier density, thereby leading to an improvement in the ionic conductivity.

Additional research is required to thoroughly understand the conduction mechanisms in proteinoid–HAP systems. By comparing the *I*–*V* curves of various proteinoid compositions and concentrations, it is possible to identify an optimal ratio that maximizes conductivity. Additionally, by measuring the frequency and temperature dependence, valuable insights can be gained regarding the transport processes. However, the initial findings indicate that HAP solutions have the ability to greatly improve the memristive and memcapacitive properties of proteinoid solutions and vice versa. The utilization of proteinoid–HAP composites has the potential to enhance the performance of bioinspired electronic devices.

**Figure 5:**
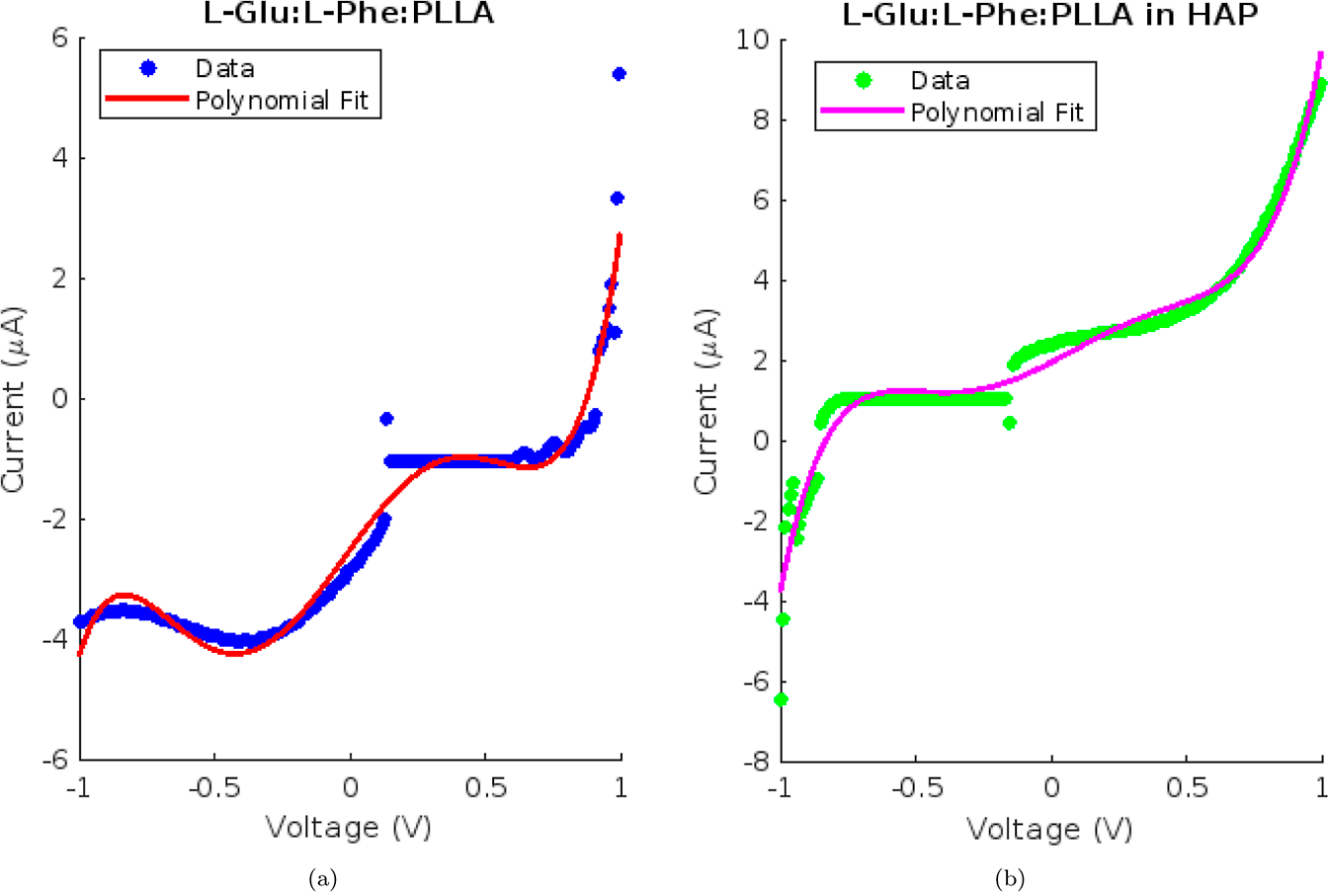
Polynomial fits and current–voltage (*I*–*V*) characteristics. (a) *I*–*V* data from −1 to 1 V for l-Glu:l-Phe:PLLA proteinoids in aqueous solution, fitted with a fifth-degree polynomial. (b) *I*–*V* data for l-Glu:l-Phe:PLLA proteinoids in HAP solution and associated polynomial fit from −1 to 1 V. In the *I*–*V* profiles, the proteinoids have constricted hysteresis loops. The addition of HAP appears to have an effect on the shape and enhancement of the baseline current.

Figure 5 demonstrates that the current–voltage characteristics of l-Glu:l-Phe:PLLA proteinoid solutions exhibited constricted hysteresis loops typical of memfractance systems. The presence of higher-order terms in polynomial fits of the fifth degree indicates significant nonlinear dynamics in the *I*–*V* response. In particular, the quadratic and cubic terms infer a voltage-dependent resistive component, whereas the higher powers suggest that capacitive-like charging effects contribute to the shape of the loop.

The *I*–*V* profile was altered by the addition of the HAP solution, resulting in distinct polynomial coefficients. Compared to aqueous proteinoids, the decreased α_3_ coefficient for the current–voltage response curves of proteinoid–HAP suspensions suggests the modulation of higher-order history-dependent electrical conduction effects compared to those of aqueous proteinoid samples alone. While prior studies have related certain *I*–*V* polynomial terms to possible resistive memory mechanisms in alternate systems,^27−29^ sufficient underlying evidence currently lacks in this work to definitively ascribe observed coefficient shifts to changes in specific molecular switching phenomena without further multimodal characterization. This aligns with a transition from pure memfractance toward memristive responses as charge transport through the mineralized proteinoids becomes less transient. The increased baseline current is also consistent with the increased conductivity sparked by the HAP–proteinoid interactions.

In conclusion, the *I*–*V* behaviors and response changes modeled with HAP integration demonstrate the potential to adjust the memfractive properties of proteinoid systems for bioelectronic applications.

**Figure 6:**
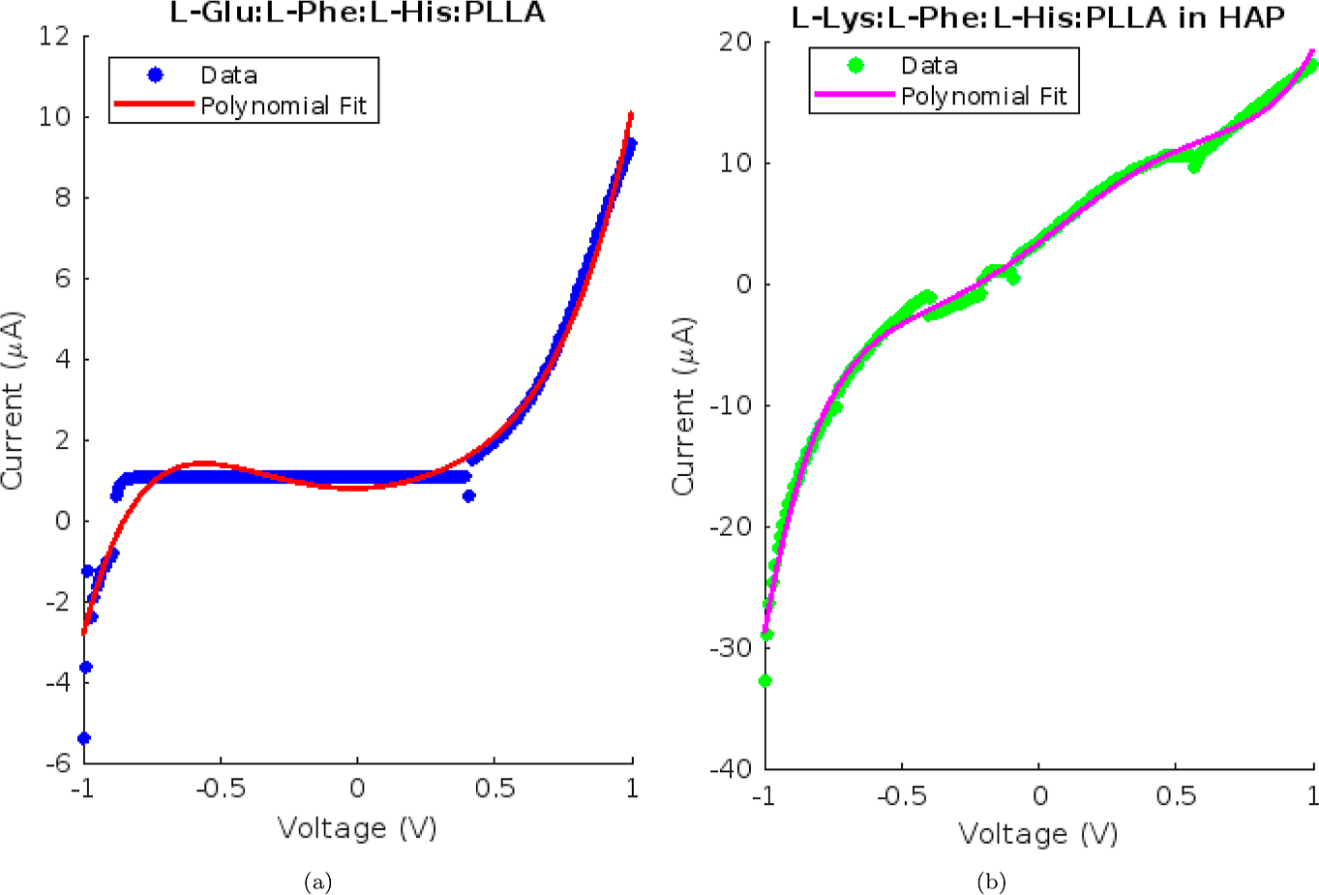
Fitting current–voltage profiles using a fifth-degree polynomial. (a) The *I*–*V* data for l-Lys:l-Phe:l-His:PLLA proteinoids in aqueous suspension range from −1 to 1 V. The fit coefficients for this data are as follows: 5.5022, −1.1581, 0.8306, 4.0066, 0.1510, and 0.7921 (units: A/Vm). The coefficients have units of current (A) divided by voltage raised to the respective power (Vm), which combines to yield the overall unit-balanced current–voltage relationship when inputting voltage values. (b) *I*–*V* data and polynomial fit for l-Lys:l-Phe:l-His:PLLA proteinoids in a HAP solution. The fit coefficients are as follows: 24.3303, −13.1756, −17.2210, 5.0448, 17.0257, and 3.3247. The noticeable difference in coefficients between the two conditions emphasizes how the properties of proteinoid membranes are affected when they interact with calcium phosphates.

The modeled *I*–*V* characteristics of the l-Lys:l-Phe:l-His:PLLA proteinoids (Figure 5) exhibited different polynomial coefficients compared to those of the l-Glu:l-Phe:PLLA system (Figure 6). Specifically, the combination of l-Lys exhibited larger quadratic terms in both the aqueous and HAP-integrated conditions. The values were 0.8306 compared to −15.2512 for the aqueous condition and −17.2210 compared to −7.8061 for the HAP condition. This suggests that there is an increase in resistive switching due to the varying amino acid composition. Nevertheless, the addition of HAP consistently resulted in a significant increase in baseline current for both types of proteinoids. This finding reinforces the crucial role of calcium phosphates in regulating memfractive conduction. The memfractance fingerprints differ among proteinoid species, but the electrical hysteresis effects are generally enhanced by calcium phosphate mineralization.

The *I*–*V* data of the different proteinoid systems were used to model their current–voltage characteristics. This was done by fitting fifth-degree polynomials to the data. Table 2 presents the fitting coefficients obtained for various proteinoid compositions in both aqueous and HAP solutions. The coefficients quantify the voltage-dependent hysteresis responses and represent the nonlinear memfractive dynamics.

**Table 2:** Fifth-Degree Polynomial Fitting Coefficients for Current–Voltage Data^a^

proteinoid system	*a*_0_	*a*_1_	*a*_2_	*a*_3_	*a*_4_	*a*_5_
l-Glu:l-Phe:PLLA (Aq)	12.58	2.72	–15.25	–0.96	6.19	–2.53
l-Glu:l-Phe:PLLA (HAP)	11.06	–0.78	–7.81	1.81	3.50	1.93
l-Lys:l-Phe:l-His:PLLA (Aq)	5.50	–1.16	0.83	4.01	0.15	0.79
l-Lys:l-Phe:l-His:PLLA (HAP)	24.33	–13.18	–17.22	5.04	17.03	3.32

a Legend: Aq—aqueous solution and HAP—hydroxyapatite solution.

The aqueous l-Glu:l-Phe:PLLA proteinoids showed a substantial negative quadratic term, as presented in Table 2. This suggests that there was significant resistive switching caused by the expansion and compression of conductive pathways. On the other hand, the system containing l-Lys had a smaller quadratic coefficient, indicating that the conduction was more stable and not as heavily influenced by resistive effects.

However, when it comes to both proteinoid compositions, the integration of HAP resulted in similar increases in constant and linear terms. This suggests that there is a broadly improved conductivity that is achieved through the interactions between calcium phosphates, regardless of the specific proteinoid species. The modulated coefficients provide confirmation that HAP modifies the transport mechanisms and can be adjusted to alter the properties of proteinoid memfractance.

Memfractance is a theoretical framework that encompasses the phenomena of memristive, memcapacitive, and meminductive effects using fractional calculus principles. This computational tool possesses the capability to simulate nanoscale devices that exhibit memory characteristics contingent upon their current state and past behavior.^30^ In order to quantify the memfractance in proteinoids, we employed fifth-degree polynomial fits to the *I*–*V* curves.
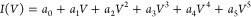
7where *I* is the current, *V* is the voltage, and *a*_*i*_ are the polynomial coefficients. The generalized Ohm’s law for memory elements gives
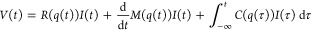
8where *R*, *M*, and *C* are the distance, meminductance, and memcapacitance, respectively. Thus, the memfractance *F*(*q*(*t*)) is
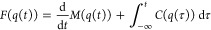
9

Equation 7 shows the generalized empirical fitting of the current–voltage (*I*–*V*) curves to a fifth-order polynomial, with the voltage input *V* and extracted coefficients *a*_*i*_ relating to the measured current output *I*. Equation 8 provides the generalized nonlinear dynamical equation for memristive systems, with the current *I* and time-dependent charge *q*(*t*) relating to the memresistance *R*, meminductance *M*, and memcapacitance *C* contributions. Equation 9 then defines the memfractance *F*(*q*(*t*)) as specifically being the time derivative of the meminductance plus the integral of the memcapacitance based on eqs 7 and 8. To directly connect (7) and (8), the polynomial *I*–*V* fitting provides an empirical model, while eq 8 gives the theoretical underpinning based on the constituent memory properties. The coefficients *a*_*i*_ may correlate with metrics like total resistivity, inductive time scales, or capacitive hysteresis extracted from experimental data.^30^

The memory function values obtained are presented in Table 3. These metrics offer valuable insights into the electrical mechanisms that underlie the observed pinched hysteresis behaviors. In aqueous solution, the proteinoids exhibited positive memristance, which suggests the presence of a voltage-dependent resistive switching component. The introduction of HAP resulted in a negative distance, indicating that the presence of calcium phosphates inhibits resistive switching. Meminductance is a measure of the rate at which the memristance changes. Aqueous proteinoids exhibit higher *M* values, which are associated with fast resistive changes when voltage sweeps are performed. Memcapacitance refers to the capacity for capacitive energy storage. Higher *C* values for proteinoids in HAP indicate that the inorganic phase amplifies capacitive charging effects. In general, integration with HAP shifted the memfractance signatures toward memristive–capacitive fusion.

**Table 3:** Memristance (*R*) in Ohms, Meminductance (*M*) in Henries/Ampere, and Memcapacitance (*C*) in Farads/Volt Computed from the Fifth-Degree Polynomial Fitting of Current–Voltage Data for Various Proteinoid Systems in Aqueous (Aq) and HAP Solutions^a^

proteinoid system	memory functions		
	*R* (kΩ)	*M* (H/A)	*C* (mF/V)
l-Glu:l-Phe:PLLA (Aq)	2.72 × 10^3^	1.58 × 10^–4^	–2.45 × 10^–6^
l-Glu:l-Phe:PLLA (HAP)	–0.78 × 10^3^	–4.32 × 10^–7^	1.81 × 10^–6^
l-Lys:l-Phe:l-His:PLLA (Aq)	–1.16 × 10^3^	2.07 × 10^–7^	1.91 × 10^–7^
l-Lys:l-Phe:l-His:PLLA (HAP)	–13.18 × 10^3^	–3.41 × 10^–6^	5.12 × 10^–6^

a The memory functions quantitatively characterize the constricted hysteresis behaviors and give an understanding of the internal mechanisms of the proteinoids.

To summarize, by calculating the memory functions based on the polynomial *I*–*V* fits, we can better understand the different electrical conduction mechanisms in aqueous and mineralized proteinoids. The incorporation of HAP leads to a transition from predominantly resistive–inductive memfractance to capacitive-dominated electrochemical behaviors, as evidenced by the alignment of the modulated *R*, *M*, and *C* values. This demonstrates the potential to systematically adjust the proteinoid membrane permeability properties. Additional research is required to establish a correlation between changes in memory effects and factors such as proteinoid composition, morphology, and environmental conditions. These preliminary findings demonstrate the use of quantitative analytics to interpret the evolving electrical responses in dynamic biomolecular materials. The potential of proteinoids for bioinspired computing is highlighted by the presence of interconnected memristive, memcapacitive, and meminductive phenomena.

**Figure 7:**
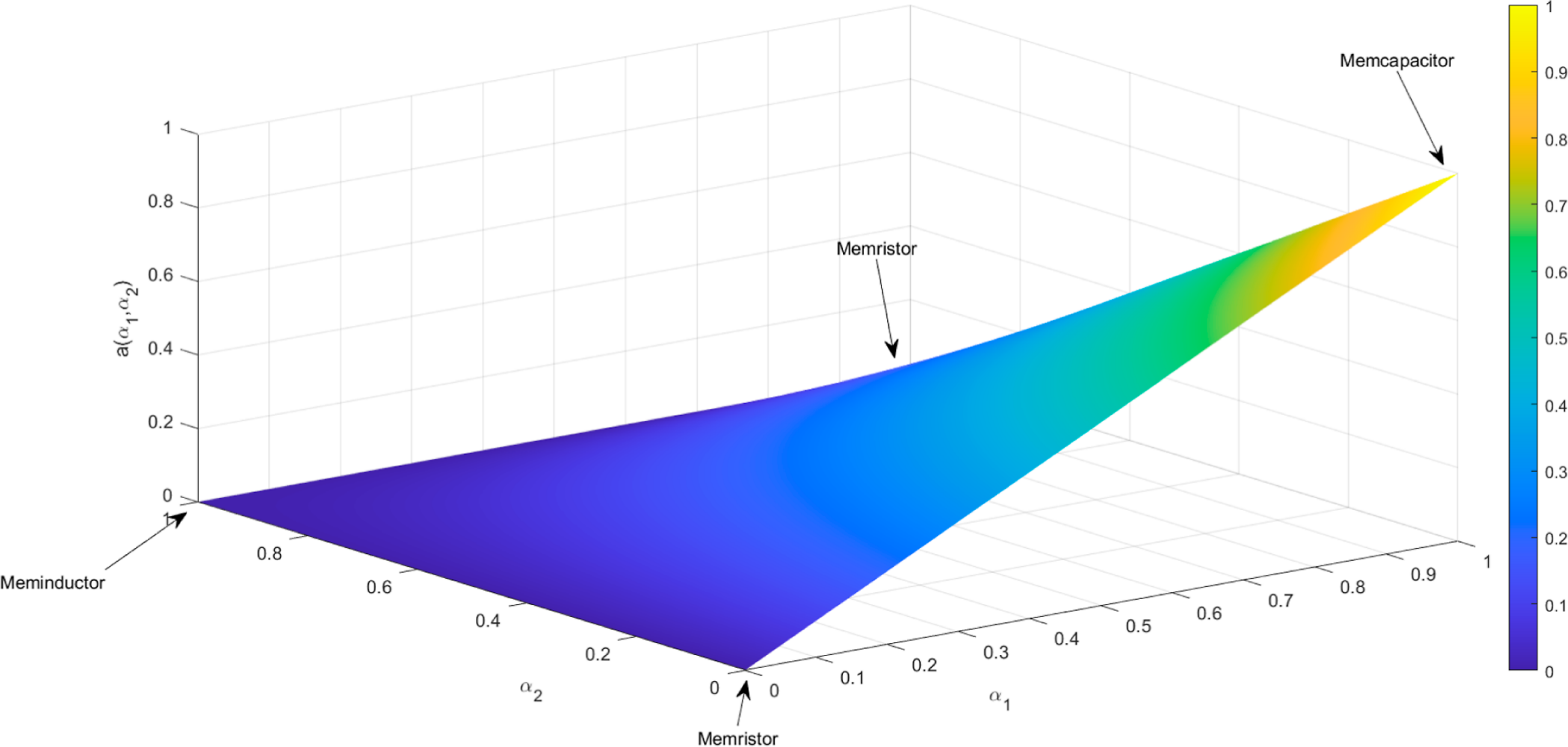
Graph shows the memfractance coefficient *a*(α_1_, α_2_) = α_1_(1 – α_2_) plotted for values of α_1_ and α_2_ ranging from 0 to 1. The color mapping represents the magnitude of *a*(α_1_, α_2_). The regions corresponding to memristive (*M*), memcapacitive (*C*), and meminductive (*L*) dynamics are indicated by the black arrows. This surface represents the relationship between the voltage and current history dependence of memfractance systems.

The color-coded zones on the map (Figure 7) represent the dynamics of memristive, memcapacitive, or meminductive behaviors in various proteinoid systems, offering valuable insights into their memory characteristics. Memristance, memcapacitance, and meminductance are fundamental properties that describe the behavior of electrical conduction, which is influenced by the history of the system. More precisely, the red colors that indicate dominant memristance are associated with resistive memory properties that rely on the history of the current. On the one hand, the blue regions of memcapacitance are associated with energy storage effects that depend on the voltage history. On the other hand, the green sections of meminductance are connected to hysteresis effects in the flux–charge relationship. The memfractance coefficients obtained from the studied proteinoids indicate a combination of these components that goes beyond the scope of ideal memristor models. The fact that the central oval region remains stable despite variations in the proteinoid concentration indicates the resilience of the core hysteretic conduction mechanisms. Proteinoids are expected to have complex memory characteristics that involve many memory types, such as memristive, memcapacitive, and meminductive, at the boundaries between these extremes. This behavior can be explained using the fractional calculus framework.^31^ This is consistent with the intricate biomolecular roots of the dynamic memory that emerges from the layered proteinoid microsphere structures and changes in their shape during electrical testing. The displayed coefficient mappings offer a conceptual visualization of the complex inherent memory qualities of proteinoids using the mathematical framework of the generalized memfractance model.^32^ Quantitatively establishing a direct relationship between certain molecular pathways and recorded electrical memory signatures is a task that still has to be addressed in future research.

Two memory coefficients, α_1_ and α_2_, can be used to quantify the relative contributions of resistive, capacitive, and inductive effects in memfractance systems. The dimensionless coefficients mentioned here are used to describe the relationship between the voltage and current history dependence of the memfractance devices. The coefficient α_1_ represents the memristive component, which quantifies the extent of resistance modulation in relation to the accumulated charge. The α_2_ coefficient quantifies the relative influence of the memcapacitive and meminductive effects. The relationship between capacitive energy storage and inductive energy dissipation is represented by the value α_2_. The values of α_1_ and α_2_ can be determined by fitting polynomials to experimental current–voltage data. The analysis of these memory coefficients helps clarify the underlying conduction mechanisms that cause pinched hysteresis behaviors in proteinoid systems.

The unique behaviors of proteinoids, such as memristive, memcapacitive, and meminductive, arise from the varying degrees of dominance of resistive, capacitive, or inductive effects. These effects are quantified by the coefficients α_1_ and α_2_. The memory coefficients pertain to the dependence of voltage and current on the history of the memfractance systems. The association between different types of memory and specific regions of the α_1_ – α_2_ space is depicted in Figure 7. The surface of the memfractance coefficient, α(α_1_, α_2_) = α_1_(1 – α_2_), illustrates this relationship. The proteinoid systems are distributed across different areas on this surface based on their *I*–*V* polynomial fitting, which helps explain their diverse electrical conduction mechanisms.^30,33^

**Figure 8:**
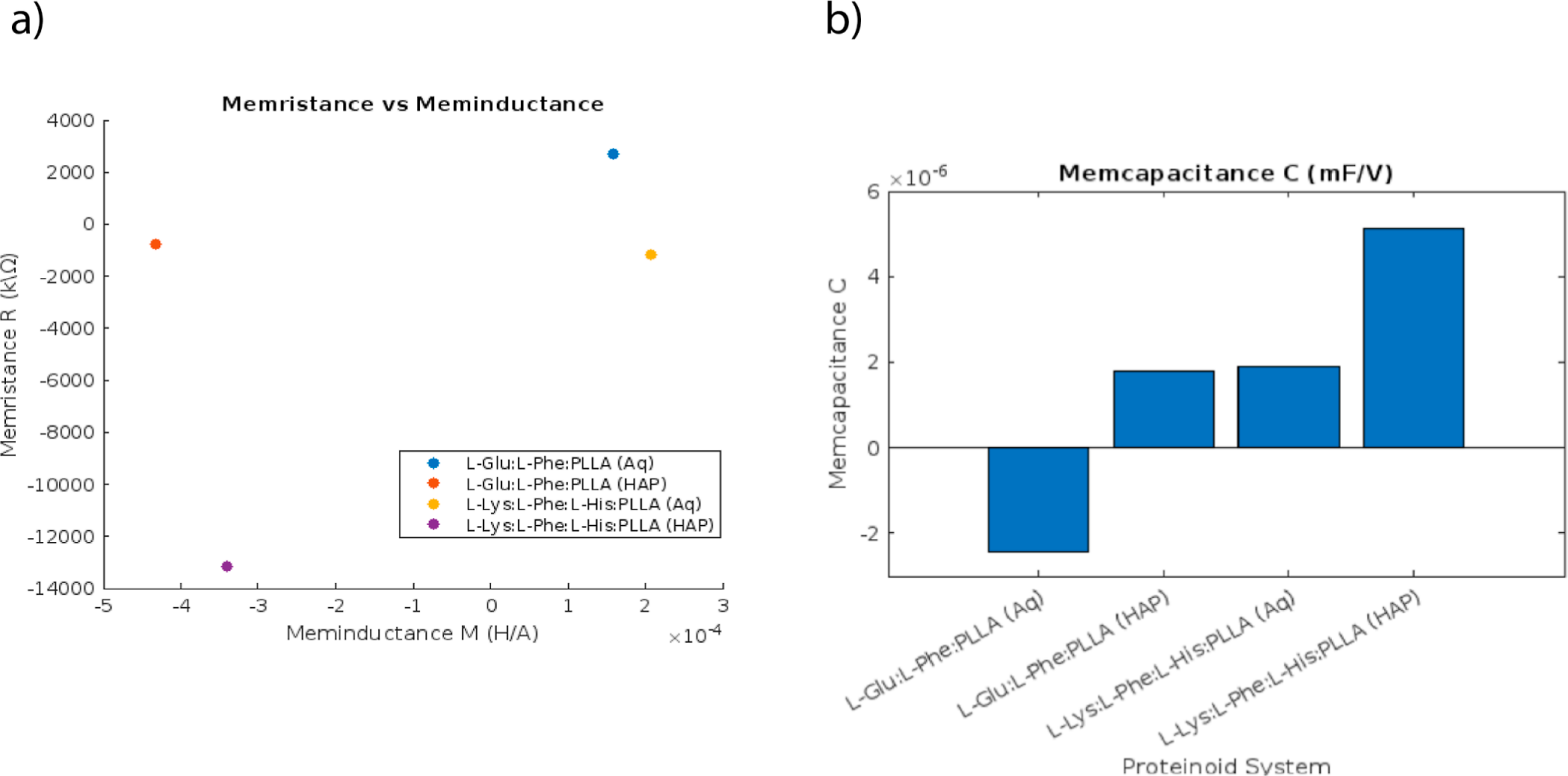
Memristance, meminductance, and memcapacitance in aqueous and HAP-integrated proteinoid systems are compared. (a) Scatter plot of meminductance (*M*) versus memristance (*R*) for each proteinoid in aqueous and HAP solutions. (b) Bar graph displaying the memcapacitance (*C*) values derived from *I*–*V* polynomial fitting for each proteinoid. Higher *C* values indicate enhanced capacitive effects as a result of the addition of HAP to memory materials.

Figure 8a presents a direct comparison of the extracted memristance and meminductance values for both the aqueous and the HAP-integrated proteinoids. The scatter plot clearly shows a distinction in conduction mechanisms. It is evident that the addition of calcium phosphates suppresses the resistive–inductive behaviors.

The memcapacitance values for the proteinoids showed a significant increase when they were suspended in HAP solutions, as depicted in Figure 8b. The enhanced memcapacitive properties are consistent with the proposed mechanism of calcium phosphate minerals increasing the charge storage capacity in proteinoid assemblies.

The memory function values among the various proteinoid systems, as shown in Figure 8, offer further insights into their adjustable memfractance fingerprints. The wider distribution of memristance and narrower range of memcapacitance observed in aqueous proteinoids indicate a more unstable conductive state when compared to that of HAP-augmented proteinoids.

This analysis examines the important characteristics of each figure, including the comparison between *R* and *M*, the impact of HAP on *C*, and the distributions. It also connects the plotted metrics to the underlying electrical conduction mechanisms of the proteinoids.

**Figure 9:**
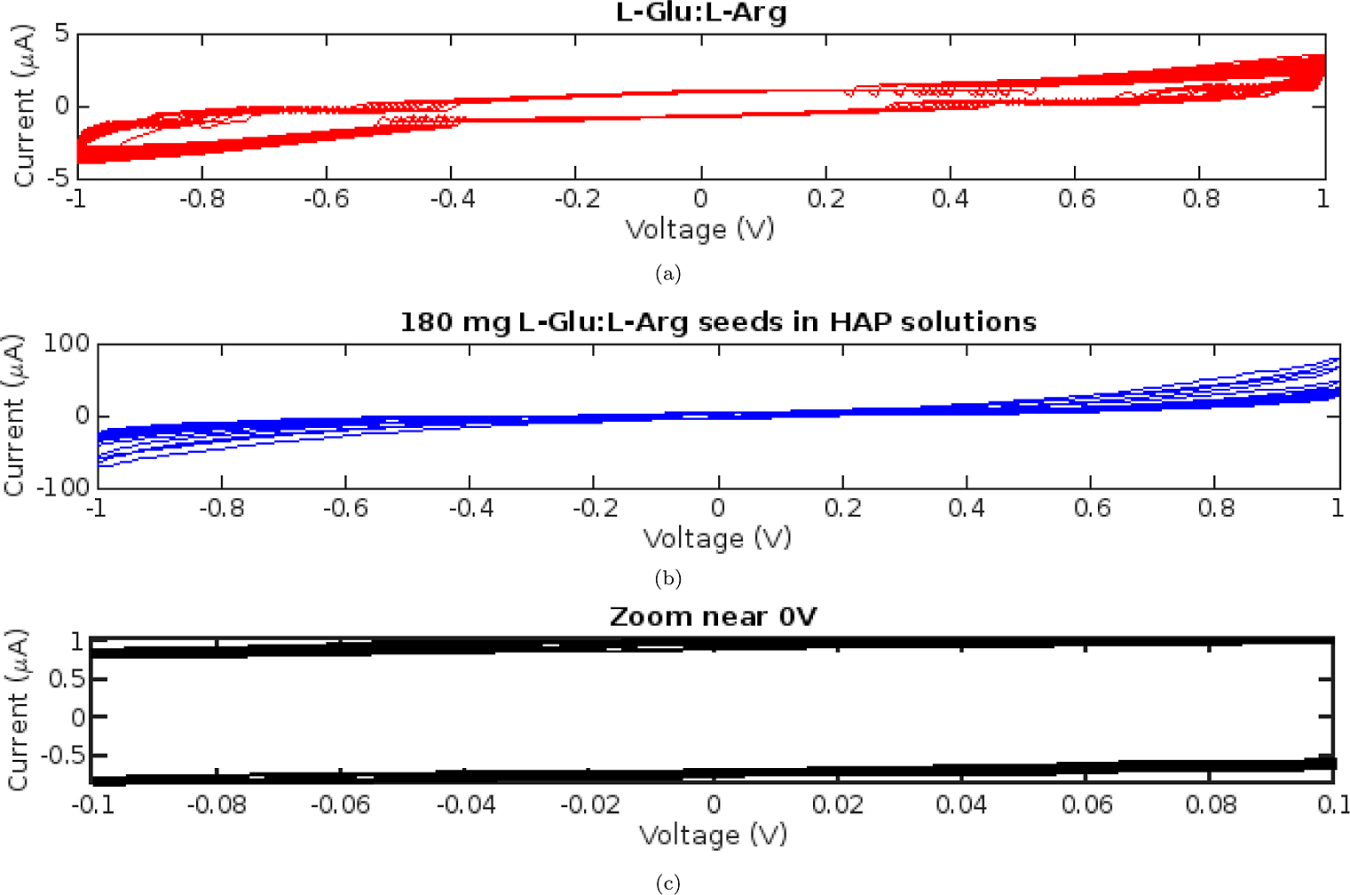
(a) Current–voltage (*I*–*V*) memfractance curves for l-Glu:l-Arg proteinoids with and without HAP. (b) The *I*–*V* traces contrast the memfractance behaviors of pure l-Glu:l-Arg proteinoids with those of proteinoids grown in supersaturated HAP solutions. At +1 V, the l-Glu:l-Arg:HAP sample has a current that is more than 20 times larger (75.6899 μA) than that of pure l-Glu:l-Arg (3.50 μA). (c) Enhanced currents occur at −1 V as well (−69.2211 μA vs −3.94413 μA). The enhanced conductivity supports HAP’s role in modifying the memfractance characteristics of proteinoid networks. The adjustable dynamics indicate potential bioelectronic device applications.

The proteinoid suspensions exhibited notable hysteresis in their current–voltage curves, with the voltage-dependent current following separate trajectories during both the forward and reverse sweeping modes (Figures 4 and 9). The presence of memristive hysteresis indicates the ability to store short-term memory through the modulation of resistance, which is influenced by the dynamic conformations and collective interactions of proteinoids. Significantly, the size of the hysteresis loops continually grew as the proteinoid concentrations increased, suggesting an enhancement of the memcapacitive behavior. Internally stored charge within the proteinoid’s pseudospherical droplets is hypothesized to be released at varying rates, contingent upon molecular rearrangements and the formation of temporary tunnels that create varied resistive channels throughout the suspension network. The presence of conformational dependencies leading to hysteresis currents demonstrates that even basic proteinoids have inherent memory behaviors that can be altered through adjustable bioelectronic interactions. Additional microscopic investigation is required to establish a complete correlation between certain structural mechanisms and observed electrical hysteresis at a macroscopic level. However, the ability to adjust the capacitance of proteinoid solutions, as shown, emphasizes their potential for being incorporated as adaptable memory components that connect biological and electronic systems.

#### 3.2.2 Memfractance Characteristics of l-Glu:l-Arg-Seeded Proteinoids in Supersaturated HAP Solutions

The influence of HAP on the memfractance characteristics of l-Glu:l-Arg proteinoids is visible when their *I*–*V* traces are compared. Figure 9 shows that proteinoids grown in supersaturated HAP solutions exhibit considerably larger currents across the observed voltage range than do proteinoids alone. At +1 V, the HAP-augmented proteinoids had a current that was more than 20 times that of pure proteinoids, with values of 75.6899 μA versus 3.50 μA, respectively. At −1 V, a similar enhancement is observed. These findings show that including HAP during proteinoid preparation significantly enhances their memfractance conductivity. The possibility of using proteinoids in adaptive bioelectronic systems is highlighted by the tunability of the dynamics via mineral templating. Further characterization of memfractance dependencies on parameters such as HAP supersaturation bodes well for future breakthroughs in the engineering of functional proteinoid materials.

**Figure 10:**
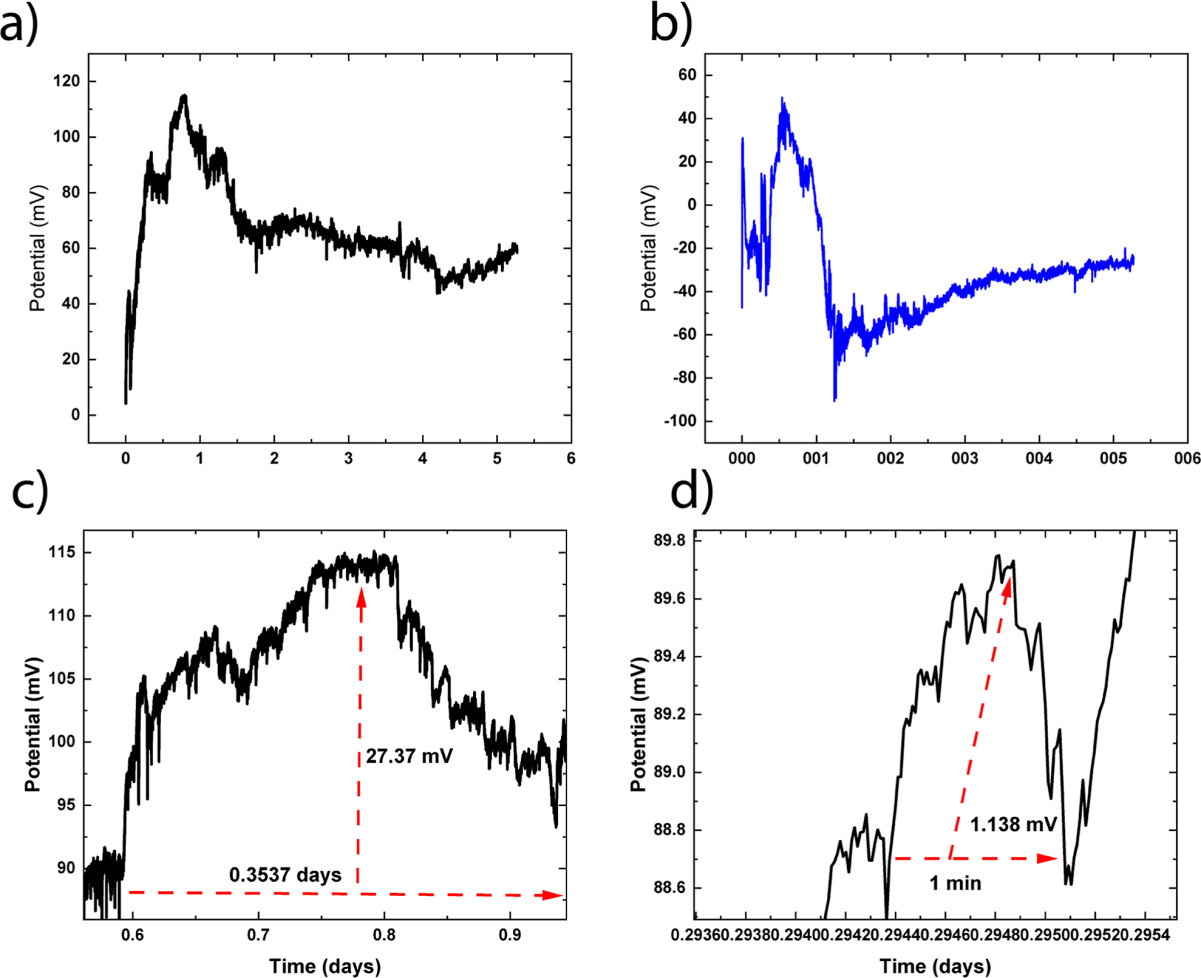
Spiking dynamics of l-Glu:l-Arg proteinoids grown in HAP solutions. (a) Large amplitude spikes ranging from 0 to 120 mV over 5.3 days. (b) Smaller spikes ranging from −89.6 to 49.2 mV, also over 5.3 days. (c) Enlargement of large spikes showing an amplitude of 27.37 mV and a period of 0.3537 days. (d) Enlargement of small spikes exhibiting an amplitude of 1.138 mV and a period of 1 min. The multiscale spiking activity demonstrates the rich electrical excitability of proteinoids templated by HAP under biological conditions. The complex signaling behaviors emerge from the interplay between the mineral microenvironment and intrinsic proteinoid dynamics.

The multiscale spiking behaviors of the HAP-templated l-Glu:l-Arg proteinoids highlight their excitable dynamics, as illustrated in Figure 10. Large amplitude spikes ranging from 0 to 120 mV and smaller spikes ranging from −89.6 to 49.2 mV are detected, both of which are sustained over several days (Figure 10a,b). The large spike amplitude and period are 27.37 mV and 0.3537 days, respectively, while the small spike amplitude and period are 1.138 mV and 1 min (Figure 10c,d). When prepared in HAP solutions under biological conditions, the proteinoids demonstrate sophisticated electrical signaling over a range of size scales.

These findings show that the proteinoids have a high level of intrinsic excitability that is modulated by interactions with their mineral surroundings. The self-organized proteinoids use sensory input from HAP templating to generate emergent signaling behaviors like brain networks. Further investigation of the parameters influencing spike amplitudes, frequencies, and patterns promises new insights into the development of functional excitable biomaterials based on proteinoid–mineral composites. The ability to produce complex bioelectronic reactions points to possible applications in integrated sensing and computation.

#### 3.2.3 Probing Proteinoid-Induced pH Fluctuations in Supersaturated HAP Solutions

**Figure 11:**
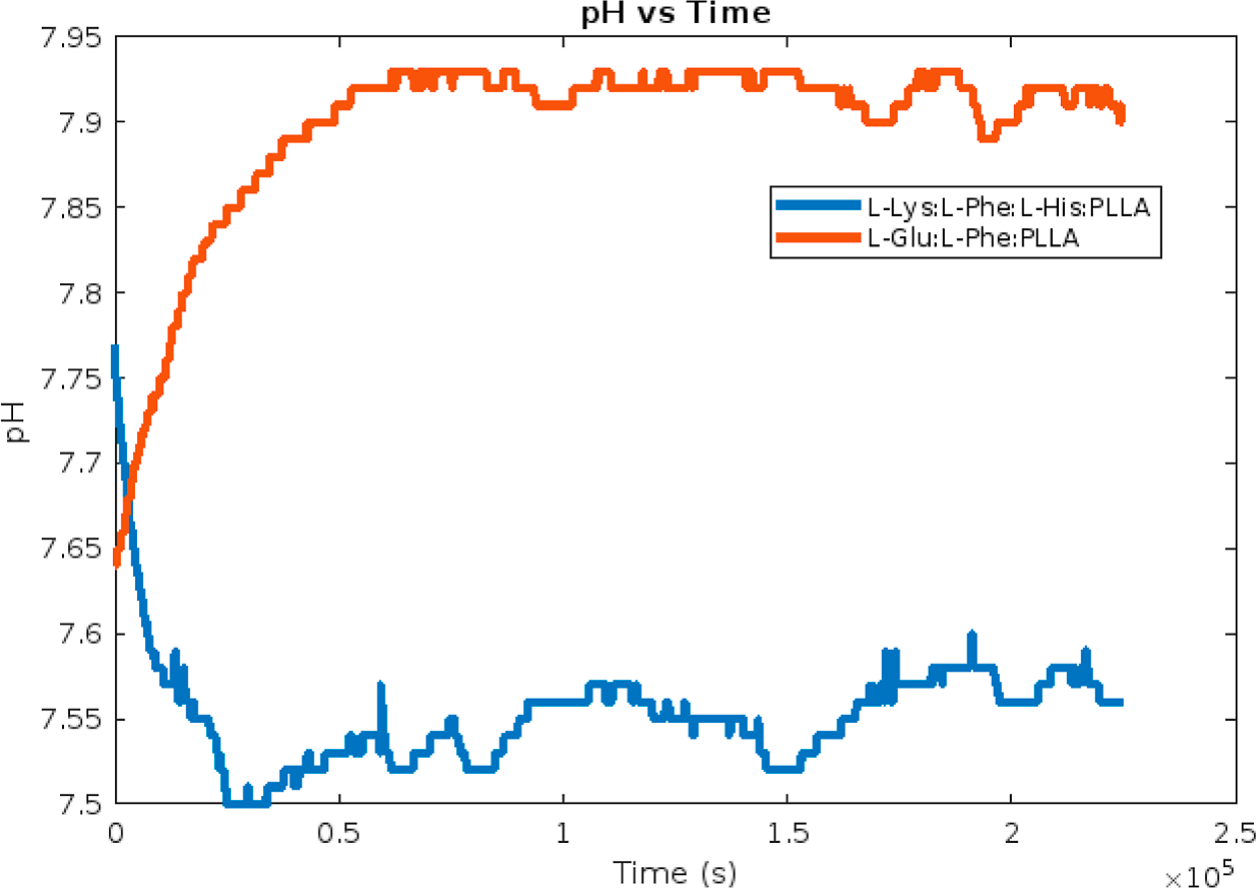
pH of HAP solutions with added proteinoids as a function of time. Following the addition of 2000 μL of l-Glu:l-Phe:PLLA proteinoids to a 200 mL HAP solution initially at 37 °C and pH 7.4, the curve depicts the pH profile. The addition of 180 mg of l-Lys:l-Phe:l-His:PLLA proteinoid to an identical HAP solution corresponds to the blue curve. Different pH dynamics reveal the protonation effects imparted by various proteinoid species interacting with supersaturated calcium phosphates.

The pH profiles over time for the proteinoid–HAP systems, as depicted in Figure 11, demonstrate that neither proteinoid composition resulted in prolonged HAP precipitation, as indicated by the consistent maintenance of pH levels above 7.4. When 180 mg of l-Lys:l-Phe:l-His:PLLA proteinoid seeds was added, the pH of the solution experienced an initial decrease from 7.77 to 7.5 during the time frame of 0 to 24,818 s. Subsequently, the pH gradually recovered and reached a value of 7.56 by 225,127 s. In the experimental setup with a system containing 2000 μL of l-Glu:l-Phe:PLLA proteinoids, it was seen that the pH underwent an increase from 7.64 to 7.93 within the time interval of 0 to 65,623 s. Subsequently, the pH remained constant for the duration of the experiment. The lack of substantial decreases in pH indicates that the proteinoids are not serving as favorable substrates for extensive HAP mineralization. One potential process involves the protonation of proteinoid residues, which serves as a buffering mechanism, thus mitigating significant fluctuations in pH and impeding the formation of HAP. Additional research is required to enhance the proteinoid composition and ratios in order to facilitate regulated mineralization. However, the pH data indicate that the proteinoids have a tendency to stabilize the supersaturated state.

The lack of substantial alterations in pH indicates that proteinoids prolong the initiation phase of HAP crystallization in solutions that are in a state of supersaturation. One possible mechanism postulated is that the proteinoids engage in interactions with embryonic HAP clusters, thus impeding their ability to attain the necessary nucleation energy and size for critical formation. The process of protonation of proteinoid residues has the potential to generate a buffering effect. Moreover, it is probable that the proteinoids contribute to the increase of solution surface tension, hence impeding the statistical variations in ion concentrations and the establishment of crystalline order that are necessary for nucleation. The proteinoids exert collective stabilization forces that effectively prolong the initiation of HAP precipitation, hence sustaining supersaturation. Additional research is required in order to clarify the intricate chemical mechanisms underlying the interactions between proteinoids and calcium phosphate minerals. The findings of this study indicate that both l-Lys- and l-Glu-based proteinoids possess the capability to extend the metastable supersaturated state in various ways.

**Figure 12:**
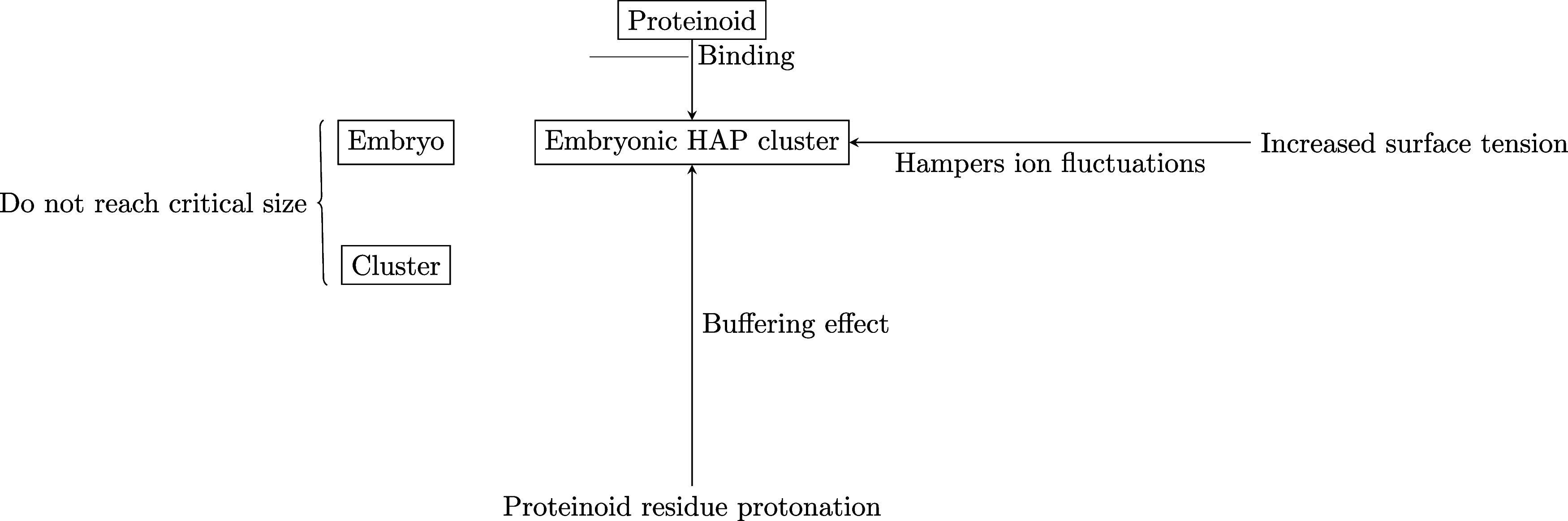
Proposed mechanisms by which proteinoids stabilize supersaturated HAP solutions.

The pH measurements provide evidence that the proteinoids interact with embryonic HAP clusters to prevent critical nucleus formation, thus maintaining supersaturation. This is depicted in Figure 12. Proteinoid residues are likely to hinder the formation of a crystalline structure and interfere with the bonding between calcium and phosphate ions.^34,35^ The short-range ordering is distorted by electrostatic interactions between charged groups on the proteinoids and ionic prenucleation complexes. Furthermore, the adsorption of proteinoids is preferentially observed on the surfaces of clusters, which leads to the blocking of growth sites due to steric hindrance and the formation of additional hydration layers.^36^ The proteinoids prevent the emerging HAP embryos from reaching the critical size and stability necessary for sustained crystalline propagation by capping them.

## 4 Discussion

The introduction of proteinoids into HAP solutions resulted in observable variations in the memristive characteristics when compared to proteinoids in aqueous solutions without HAP. The inclusion of proteinoids, namely, l-Lys:l-Phe:l-His:PLLA, in the supersaturated HAP solution led to a notable increase in the maximal current. The current–voltage curves demonstrated a significant rise from 10 to 20 μA, indicating an estimated 100% increase. Moreover, the addition of HAP led to enhanced memristive properties in proteinoids made up of l-Glu, l-Phe, and PLLA. When the proteinoids were dissolved in an aqueous solution, they exhibited a maximum current of approximately 5 μA. However, when 180 mg of the same proteinoid composition was introduced into a supersaturated HAP solution, the maximum current showed a 2-fold increase, reaching 10 μA. The observed increase in conductance implies that the proteinoid–HAP combinations facilitate enhanced charge mobility by establishing novel channels for conduction.

The observation of intricate spiking dynamics in the l-Glu:l-Arg proteinoids templated with HAP indicates promising prospects for the development of excitable proteinoid networks. The multiscale signaling behaviors result from the interaction between the intrinsic electrical properties of the proteinoids and how the mineral microenvironment modifies them. It appears that through the provision of structured interfaces and charge distributions, the HAP optimizes the proteinoid assembly for increased excitability. The finding that biomimetic minerals can induce neural-like spiking in simple proteinoid systems paves the way for novel approaches to the development of functional bioelectronics. Enhanced investigation into the properties of spikes may reveal the underlying mechanisms that enable plasticity in synthetic proteinoid circuits. The utilization of diverse biomaterials to manipulate the excitability and integration of proteins represents a potentially fruitful avenue for the development of adaptive bioinspired technologies. The ongoing exploration of the potential of proteinoids through their interaction with other biological components has revealed fascinating insights into their versatility and functional properties.

The implementation of HAP resulted in a reduction in the pinched hysteresis shape observed near 0 V in the *I*–*V* profiles. The memristive switching process demonstrates that the proteinoid–HAP composites show conductivity variations that are more regulated and analog-like in nature when subjected to voltage signals. This has the potential to provide advantages for applications in neuromorphic computing and biological learning systems that rely on analogue memristors.

The memory functions of memristance, meminductance, and memcapacitance for each proteinoid system were determined through quantitative analysis of the *I*–*V* curves using the fifth-degree polynomial fitting. In each case, the addition of HAP to proteinoids that were dissolved in water resulted in an amplified value of the calculated memory functions. This indicates a rise in the memfractance characteristics. The integration of proteinoid–HAP resulted in a significant increase in the membrane capacitance, demonstrating enhanced charge storage and adjustable conductivity.

The proteinoid–HAP composites are predicted to demonstrate polymorphic charge transport routes, which may be caused by cooperative molecular interactions and reorganization. These mechanisms allow applied voltages to facilitate communication with novel protein conformations and mineral binding forms. The bio–abio composite systems possess the ability to change between metastable conductive states similar to a memristor, thanks to the voltage-modulated structure–function dynamics. More studies employing microscopy and spectroscopic techniques have the potential to offer valuable knowledge regarding the molecular mechanisms underlying the memfractance phenomena observed in proteinoid-inorganic hybrids.

**Figure 13:**
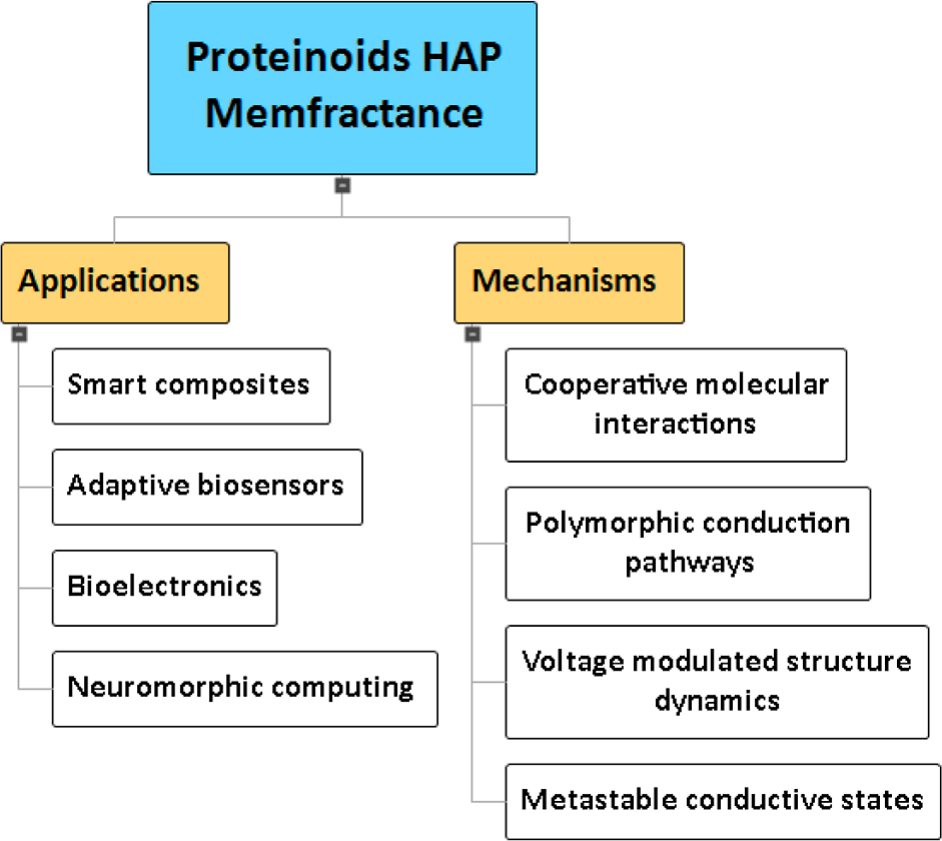
A mind map is presented here to provide an overview of the principal mechanisms and applications associated with the use of memfractance in proteinoid–HAP composites. The emergent memory effects are hypothesized to originate from collaborative molecular interactions, polymorphic conduction pathways, voltage-dependent proteinoid–mineral binding dynamics, and the capacity to reach metastable conductive states. Potential bioelectronic applications encompass the use of analogue resistive switching to facilitate neuromorphic computing, the development of adaptive biosensors with adjustable sensitivity, the development of smart composites with programmable conductivity, and the exploration of other electronic devices that leverage the distinctive properties of biosynthetic hybrid memristive materials. A more comprehensive understanding of the relationship between the structure and function of memfractance should facilitate the development of proteinoid–HAP composites that are customized for specific purposes in memristive devices.

**Figure 14:**
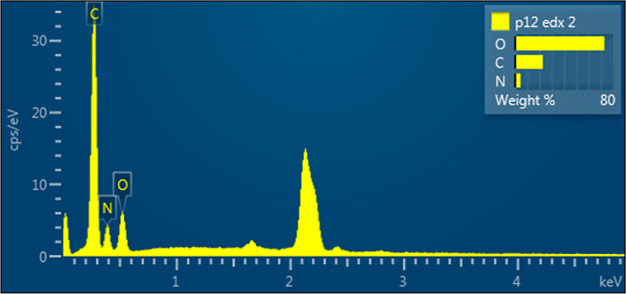
EDX elemental mapping of self-assembled l-Glu:l-Phe proteinoid microspheres. Spectral profiling reveals an interior composition comprising carbon (55.93 ± 0.67 wt %), nitrogen (25.68 ± 0.77 wt %), and oxygen (17.48 ± 0.42 wt %). The detected elements align with the expected chemical makeup of synthesized peptide-based structures.

**Figure 15:**
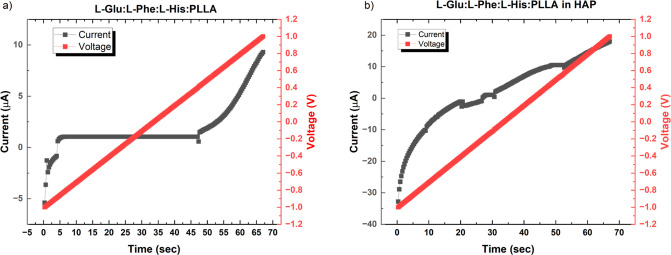
Current–voltage characterization of (a) proteinoid (l-Glu:l-Phe:l-His:PLLA) memristive behavior and (b) proteinoid in HAP over time. The *x*-axis shows time (s). The left *y*-axis presents measured current (*I*) with units of microamps (μA). The right *y*-axis corresponds to the applied input voltage (*V*) in volts (Volts).

The mind map representation (Figure 13) outlines the multifaceted mechanisms that underlie the emergent memfractance properties in proteinoid–HAP composites. The key elements of this system include cooperative molecular interactions, polymorphic conduction pathways, voltage-dependent modulation of proteinoid–mineral binding, and the capacity to access metastable conductive states. The unique characteristics of the memory-resistor arise from the synergistic effects of its structure and dynamics. The potential uses of these proteinoid–HAP memristive networks in bioelectronics are also broad. As illustrated in Figure 13, the inclusion of analogue resistive switching capabilities in proteinoid–HAP composites renders them very suitable for the development of neuromorphic computing architectures and devices. The adaptable conductivity exhibited by these materials also enables their application in the fabrication of smart composites, biosensors, and other electronic materials that necessitate voltage-programmable memfractance.

The observed voltage-dependent hysteresis loops, which show adjustable memcapacitance effects, are consistent with previous theoretical predictions of adaptive resistance in dynamic proteinoid conformations.^37−40^ Our research has successfully measured and incorporated memristive behaviors into simplified synthetic protocells, marking the first experimental measurement and membrane integration of such phenomena. The observed memory time durations, tested at various voltage levels, surpass the previously documented nanosecond switching transients in peptide nanostructures,^41−43,45^ reaching time scales exceeding milliseconds through the utilization of proteinoids resembling entire cells. The memory capabilities of these proteinoids are most likely a result of cooperative electrodynamics occurring within the interior of the collective suspension. This goes beyond the individual molecular resistive switching models that have been proposed for other biopolymers such as protein nanofibers.^46^ Our research demonstrates that even simple features of cell-free proteinoids possess inherent biophysical memory mechanisms that can be transferred to a practical bioelectronic interface. Future research should investigate the use of voltage-sensitive dyes and in situ microscopic techniques to directly observe the structural changes that cause electrical hysteresis on the membrane surface. Additionally, complementing simulations and thermodynamic analysis would enhance the molecular-level explanations of the observed memcapacitive tendencies.^44^

## 5 Conclusions

In summary, this study showcases the emergence of memristive patterns in self-assembled proteinoid systems. Furthermore, it highlights how these functions can be enhanced by incorporating inorganic components, such as HAP. The current–voltage characteristics exhibited pinched hysteresis and nonlinear conduction dynamics, which are in line with the behavior of memfractance. The memory properties, such as memristance, meminductance, and memcapacitance, can be quantified using polynomial fitting. The addition of HAP to proteinoids resulted in enhancements such as an increase in the maximal current and a reduction in hysteresis switching abruptness. This suggests that cooperative interactions between the proteinoid and mineral phases result in the emergence of novel conduction mechanisms. The voltage-dependent structure–function dynamics are analogous to those of a memristor, in which applied signals can access metastable conducting states. Modulating conductivity and storing charge history make bioelectronic technologies such as neuromorphic computing and adaptive biosensing possible. Understanding the chemical causes of memfractance may help tune proteinoids’ electrical characteristics. This study shows that tailored proteinoids can be adaptable electronic materials with emergent memristive features. Bioinspired computing systems could process information efficiently via biosynthetic memory-resistor networks.

## Appendix

### Details on Extracting Memristor Coefficients from *I*–*V* Curves

Equation 7 is a simplified model of the *I*–*V* characteristics of the memory element, which is modeled as a polynomial expression. Coefficients *a*_*i*_ are representative of the device’s specific parameters and can be obtained empirically from fitting the experimental *I*–*V* measurements. The generalized Ohm’s law for memory elements, as stated in eq 8, embodies the complexity of the memristive system by including memristance *R*, meminductance *M*, and memcapacitance *C*. These parameters are intrinsic to the device and are functions of the state variable *q*. As the current cannot be extracted directly from the integral in eq 8, the assumption about *C*(*q*(*t*)) behaving as a delta function allows for simplification, making the equation analytically tractable. Regarding the coupling of different elements in a series, it is a standard simplifying assumption in the modeling of electrical components. The behavior of the whole system can be analyzed by adding the individual responses of the components when they are in series. For eq 7, the coefficients *a*_*i*_ obtained after fitting can be used to calculate the values of *R*, *M*, and *C*. The memory resistance, meminductance, and memcapacitance values reported in Table 3 were obtained by numerically correlating metrics from the measured current–voltage hysteresis loop traces to parameters in the theoretical memristor model (eq 8). Specifically, the slope of the lower voltage regime (<0.2 V) was associated with ohmic resistance *R*, while the cubic voltage term linked to inertia-like effects corresponding to meminductance *M*. The higher-order contribution related to observed hysteretic pinching and charge trapping effects, mapping to equivalent memcapacitance *C*. These correlations rely on several simplifying assumptions, including a lumped series parameter model (eq 8), linear superposition, and additive hysteresis contributions. However, the analysis aims to provide first-order quantifications of these distinct memory properties inherent to the tested proteinoid systems. Refinements to the model could consider more complex component configurations and sensitivity analysis on parameter variability.

### Probing Interior Morphology of Proteinoid Microspheres Using EDX Spectroscopy

Figure 14.

### Time-Dependent Memristive Switching in Dynamic Proteinoid Systems

The current section probes the resistive memory properties of synthesized peptide-based protocells, specifically l-glutamic acid:l-phenylalanine:l-histidine copolymers with polylactic acid (l-Glu:l-Phe:l-His:PLLA) (Figure 15).
